# Bilateral papilledema in a child with osteogenesis imperfecta

**DOI:** 10.1186/s40662-016-0056-4

**Published:** 2016-10-17

**Authors:** Selam Yekta Sendul, Cemile Ucgul Atilgan, Semra Tiryaki, Dilek Guven

**Affiliations:** 1 Department of Ophthalmology, Sisli Hamidiye Etfal Training and Research Hospital, Etfal Street, 34280 Istanbul, Sisli Turkey; 2 Department of Ophthalmology, Ulucanlar Eye Training and Research Hospital, Ulucanlar street, 06030 Ankara, Altindag Turkey

**Keywords:** Optical coherence tomography, Osteogenesis imperfecta, Papilledema, Visual field defect

## Abstract

**Background:**

To present a female child patient with osteogenesis imperfecta who had bilateral papilledema.

**Case presentation:**

A twelve-year-old girl with osteogenesis imperfecta was referred to our clinic. Bilateral best corrected visual acuity of the patient was 5/10 (corrected with +3.50 for right eye, +5.00 for left eye) with a standard Snellen scale at a distance of a 6 m. Anterior chamber, iris and lens examination of both of her eyes were unremarkable. In her fundus examination, bilateral stage 2 papilledema and the wrinkles in papillomacular area were noticed. Optical coherence tomography images revealed the macular pucker and thickening in the retinal nerve fibre layers of both eyes. Computed tomography images revealed that there were ossifications in the optic chiasma and occlusion in all periorbital sinus areas.

**Conclusion:**

Osteogenesis imperfecta is a rare, autosomal dominant connective tissue disorder characterised by bone fractures, deafness and blue sclera. We would like to draw attention to the clinical course of our patient with computed tomography, optical coherence tomography and visual field findings.

## Background

Osteogenesis imperfecta (OI) is a heritable disease of the connective tissue characterised by lower bone mass, bone fragility and skeletal deformities. The major health consequences in OI arise from functionally compromised bone. Short stature, blue sclera, laxity of ligaments, non-union of fractures, keloid and hyperplastic callus formation are additional but variable symptoms [1].

A typical ocular finding of OI is blue sclera [2]. Additional ocular findings are the decreased ocular rigidity [3], refractive errors (myopia, hyperopia, astigmatism), glaucoma, keratoconus, keratoglobus, corneal opacity, small corneal diameter, corneal thinning, cornea plana, congenital Bowman’s layer agenesis, vitreous and retinal haemorrhage, choroidal neovascularization, retinal detachment, optic neuropathies and optic atrophy [4–12]. The study was conducted in accordance with the tenets of the Declaration of Helsinki by obtaining written consent from the parents as well as the patient.

## Case presentation

**Fig. 1:**
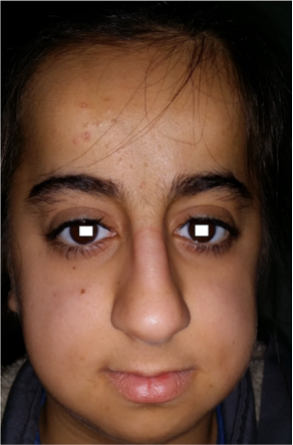
Typical facial appearance of twelve-year-old female patient with osteogenesis imperfecta

**Fig. 2:**
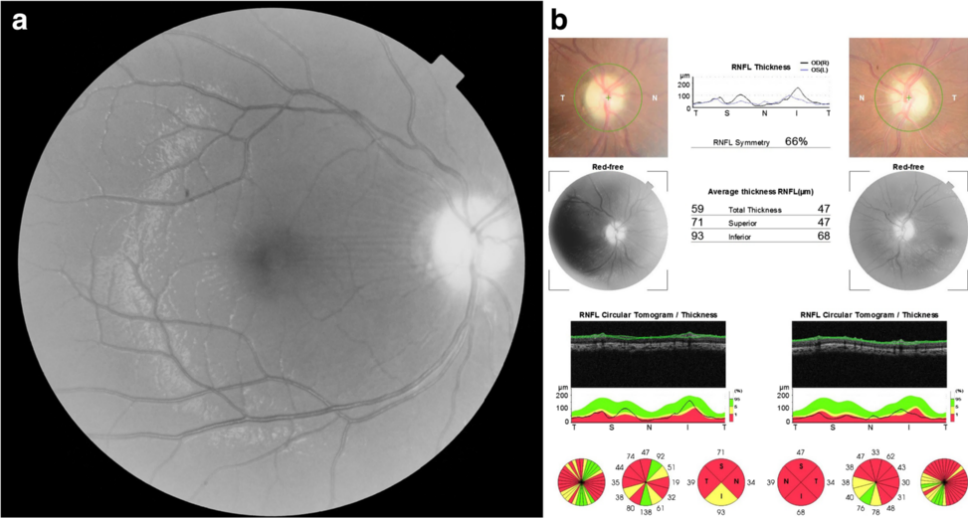
**a** The picture shows stage 2 papilledema and wrinkles in the papillomacular area during her fundus examination. **b** In her fundus examination after 5 months, the picture shows the bilateral optic discs pallidness and the distinct thinnings in her both retinal nerve fibre layers

**Fig. 3:**
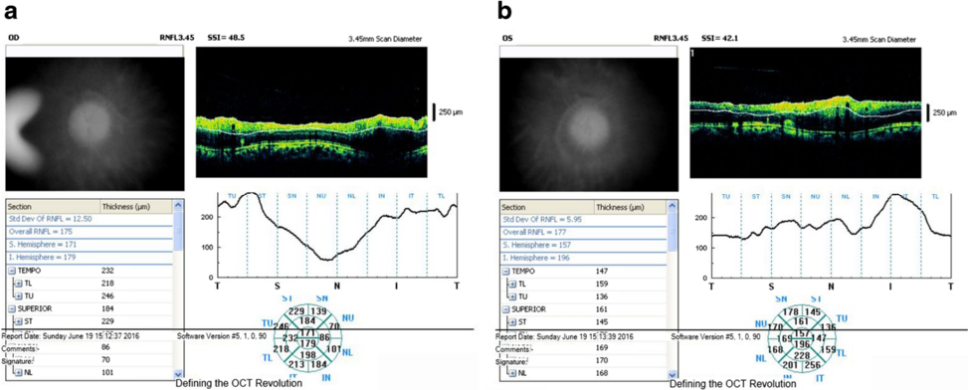
In her initial retinal nerve layer analysis, there seemed to be a thickening of both retinal nerve fibre layers due to bilateral papilledema. (**a** Right, **b** Left)

**Fig. 4:**
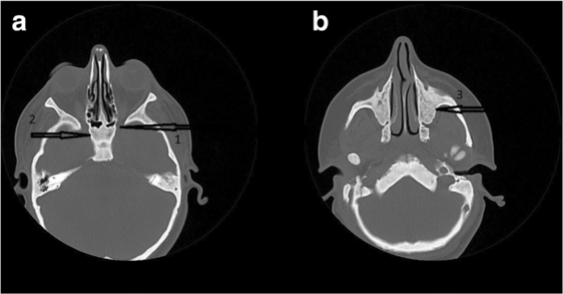
These axial tomography photographs show an ossification in the optic chiasma (**a**) and occlusion in all periorbital sinus areas (**b**)

**Fig. 5:**
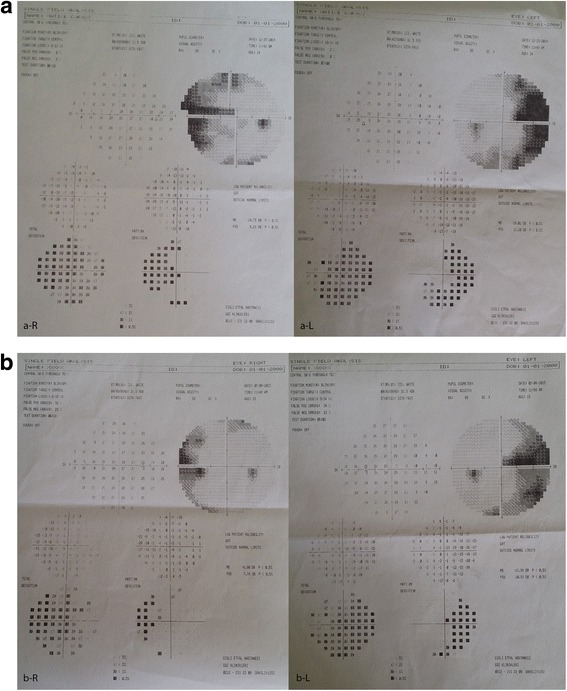
**a**, **b** Bitemporal partial hemianopia is seen in her Humphrey visual field examination. Enlarged blind spot in the papilledema period is remarkable. **a** Bitemporal partial hemianopia and enlarged blind spots in papilledema period of right and left eyes. (R: Right, L: Left). **b** After 5 months, while enlarged blind spots were improved along with decrease in papilledema, bitemporal partial hemianopia became more obvious. (R: Right, L: Left)

A twelve-year-old female patient with OI was referred to our clinic for routine eye examination from the genetic department. Bilateral best corrected visual acuity of the patient was obtained – 5/10 (corrected with +3.50 for right eye, +5.00 for left eye) – with a standard Snellen scale at a distance of 6 m. She had growth retardation and typical facial appearance compatible with OI, such as triangular shaped face, large skull, a prominent nose, elongated columella of the nose, smaller maxilla, slightly larger mandible (Fig. 1). Anterior chamber, iris and lens examination of both her eyes were unremarkable. Neither of her eyes had blue sclera. Both glob measurements measured by Hertel’s exophthalmometer were 21 mm. Bilateral corneal diameters were 10 mm at vertical axis and 9.5 mm at horizontal axis. Bilateral intraocular pressures were 12 mmHg with Applanation tonometry. Examinations of motolities of both her eyes were normal. Central corneal thicknesses of her right and left eyes with ultrasound corneal pachymetry were 500 and 510 microns, respectively. In her fundus examination, bilateral stage 2 papilledema according to Frisen Scale and wrinkles in papillomacular bundle were noticed (Fig. 2a). Bilateral colour vision examinations by Ishihara plates were normal. Initially, optical coherence tomography (OCT) images revealed the macular pucker and thickening in the retinal nerve fibre layer (RNFL) of both her eyes (Fig. 3a, b) but after 5 months when the papilledema subsided, the distinct thinning in both RNFL was obvious (Fig. 2b). Computed tomography (CT) images revealed that there were hyperostosis in the skull base, maxillofacial and frontal bone structures. In addition to extreme hypoplazic appearance in the sphenoid sinus, the other periorbital sinuses were not monitored in CT images due to their abnormal ossification (Fig. 4a, b). In her visual field examination using the Humphrey field analyser, we noted remarkable enlarged blind spot and bitemporal partial hemianopia (Fig. 5a). As the papilledema regressed spontaneously and neurological examination of the patient was normal, we decided to follow up our patient with OCT and visual field tests without treatment. In her last control examination, bilateral best corrected visual acuity was 6/10. Anterior segment examinations were normal. Bilateral colour vision examinations using Ishihara plates were normal. Bilateral optic discs were pallor (Fig. 2b). RNFL analyses of both her eyes still revealed distinct thinning (Fig. 2b). In her visual field examination, despite the improvement in the enlarged blind spot, bitemporal partial hemianopia was still remarkable (Fig. 5b).

## Discussion

Osteogenesis imperfecta is a polygenic disease with a variable phenotype spanning mild to lethal. The majority of mutations associated with OI occur in Type 1 collagen encoding genes (COL1A1, COL1A2) and give rise to an autosomal dominant form of the disease. Novel mutations in seven other genes involved in collagen assembly and processing and in two genes involved in cellular differentiation have recently been associated with autosomal recessive forms of OI [1].

In 1979, Sillence and Danks used clinical, radiographic and genetic characteristics to distinguish four groups of patients with OI [13]. Type 1 typically has the mildest musculoskeletal phenotype with common fractures but rare bony deformities and possess the clearest blue scleral hue. Type 2 is the most severe form of OI and is typically lethal during the perinatal period. Individuals with type 3 OI have progressive bone deformations and short stature with blue sclera that fades with age. In those with type 4 OI, bone involvement is variable, dental abnormalities are common, and the sclera is normal or greyish in colour. While our patient’s findings were most consistent with type 4, genetic confirmation of the diagnosis was unavailable.

The measures of incidence and prevalence vary among studies but composite evidence suggest that the incidence is probably just over 1 out of 10,000 births and prevalence is a little less, which takes into account the perinatal deaths of those with lethal forms of OI. OI has been reported throughout the world with no selection of race or gender. It could be estimated that 0.008 % of the world’s population has OI. In other words, 500,000 persons worldwide would have been afflicted with OI [12].

Patients with OI could have some eye disorders. The present findings of our patient in her initial consultation were low vision, refractive errors (hyperopia), papilledema, macular wrinkle in the papillomacular bundle. We thought that the causes of low vision in our patient were due to both macular wrinkles and amblyopia because of the absence of wearing glasses at a younger age. On the other hand, the interesting point of this case for us was bitemporal partial hemianopia, which emerged together with papilledema and then persisted. Especially in case of bilateral papilledema, the reasons that cause an increase in intracranial pressure should be ruled out. As the neurological examination and intracranial pressure of our patient were normal, we thought that bilateral papilledema and visual field defects were most probably due to abnormal ossifications and microfractures in the optic channel and optic chiasma. While the abnormal ossification that cause the local pressure to optic nerve head may result in papilledema, the compression to optic chiasma may result in visual field defects.

Papilledema related to OI is a rare complication. In addition to our case, there is a case report with optic atrophy in the literature from Russia [14].

## Conclusions

As the eye conditions are common in the OI population, these patients should have eye examinations annually, or at any time new visual symptoms may arise. As with our patient, serious symptoms such as papilledema can even be detected in a routine eye examination.
